# Augmented Reality and Plastic Surgery Training: A Qualitative Study

**DOI:** 10.7759/cureus.19010

**Published:** 2021-10-24

**Authors:** Lilli Cooper, Asmat H Din, Edmund Fitzgerald O'Connor, Paul Roblin, Victoria Rose, Maleeha Mughal

**Affiliations:** 1 Plastic and Reconstructive Surgery, Guy's and St Thomas' NHS Foundation Trust, London, GBR; 2 Plastic Surgery, Guy's and St Thomas' NHS Foundation Trust, London, GBR

**Keywords:** augmented reality, covid-19, webinar, training, technology

## Abstract

Background and objective

The coronavirus disease 2019 (COVID-19) pandemic has led to many challenges in face-to-face teaching and training in plastic surgery. However, it has also proved to be an incubator for many technological solutions. Augmented reality (AR) platforms may offer a safe, equitable, and efficient means to provide training in plastic surgery. This study aimed to explore the user's experience of AR as an educational intervention during the COVID-19 pandemic in the United Kingdom (UK).

Materials and methods

The Proximie® AR platform (Proximie Limited, London, UK) has been in use in a UK plastic surgical department for facilitating webinars, visual libraries, and streamed procedures. The experience of a range of trainers and trainees was qualitatively explored through 10 individual interviews. Data-emergent theme analysis was also performed.

Results

AR was well-received in the context of COVID-19 and training in general as a means to enable theatre access, and visual revision, remotely. The potential for its use in remote coaching and telementoring was also discussed. Recommendations were made by the users to optimise the experience both from the trainer and learner perspectives. Data were presented pertaining to the following themes: surgical AR as a substitute for hands-on learning; surgical AR and theoretical learning; considerations specific to streamed procedures using Proximie®; considerations in the use of technology in general.

Conclusion

Harnessing novel technologies in surgical education offers an exciting opportunity, fast-tracked by COVID-19, but applicable beyond it. Though this study includes a small sample size, its findings suggest that AR platforms may offer a uniquely interactive remote educational experience in surgical training. Strategies and suggestions for its use are discussed, as well as broader considerations in using technology in surgical education.

## Introduction

The coronavirus disease 2019 (COVID-19) pandemic has introduced many challenges in the delivery of plastic surgical training in the United Kingdom (UK) [1,2]. Measures such as trainee redeployment, decreased onsite footfall, and cancelled elective work have compromised longitudinal training relationships; the use of personal protective equipment and hospital protocols have reduced theatre turnover, and consultant-only operating has been encouraged [2]. Additionally, distancing measures have resulted in the cancellation of normal departmental teaching, national and international conferences, as well as meetings and exams [2].

Many different technologies exist that help to overcome these challenges and are being used successfully in medical and plastic surgical training, including augmented reality (AR) platforms [2]. AR constitutes a technology that superimposes a computer-generated image on a user's view of the real world, thereby providing a composite view. Our department has employed a Health Insurance Portability and Accountability Act (HIPAA)- and General Data Protection Regulation (GDPR)-compliant, cloud-based AR platform with multiple interactive functions (Proximie®; Proximie Limited, London, UK) for webinars and operative live-streaming (with appropriate permissions) to mitigate some of these issues. This study aimed to analyse the quality of the learning experience for trainees and trainers when using AR for learning, to identify any barriers to its use, outline potential improvements, and share lessons learnt along the way.

## Materials and methods

We used a loupe-mounted camera on the operating surgeon, in addition to one or two further cameras on microscopic or macroscopic views, to stream operative procedures to the audience on their personal devices via up to four screens, through the Proximie® platform. They could use integrated AR tools to point, demonstrate, overlay, or type on the view visible on a screen in the theatre, as well as the two-way audio system. Typically, one consultant acted as the lead operator; a second consultant assisted and wore a headset to interact with those watching, talking them through key parts of the procedure, and taking questions. A third consultant acted as remote moderator for each session, guiding the theoretical discussion, sharing images and papers, and providing continuity of teaching, to enable the operating surgeons to focus solely on the operating procedure, as required.

Additionally, by using a purposive sampling technique, we conducted interviews with various members of the plastic surgery team, including both men and women, members of different seniority levels, trainers and trainees, those in clinical management and formal educational positions, digital natives and immigrants (born after and before 1980) [3], and those working on-site and remotely. Ten participants were invited to be interviewed, and 10 structured individual interviews were iteratively conducted by telephone, until theoretical saturation was reached [4]. Funnel questions were designed along pre-designed themes based on the literature, with broad stems and probes, where required. Participants were asked to reflect on their experience of using surgical AR as a trainee, trainer, or both, its advantages and limitations within the setting of COVID-19 pandemic and beyond, and lessons learnt. The stems and probes used for the interviews are shown in Table 1.

All interviews were recorded, transcribed verbatim using an electronic transcription device (Sonix, Inc. San Francisco, CA), checked word-by-word, and de-identified prior to data emergent theme analysis. Following the grounded theory, we engaged in two analytical stages of coding: initial and focused. Initial coding was employed after reading and re-reading the interview transcripts line-by-line to ensure adequate immersion and identify overarching ideas. The initial codes were refined and developed in the subsequent round of focused coding using NVivo software (1.2, QSR International, Doncaster, Australia). The refined codes were grouped into four distinct categories:

1. Surgical AR as a substitute for hands-on learning

2. Surgical AR and theoretical learning

3. Considerations specific to streamed procedures using Proximie®

4. Considerations in the use of technology in general

**Table 1:** Pre-designed interview funnel questions with stems and probes AR: augmented reality; COVID-19: coronavirus disease 2019

Topics	Stem question	Probe layer 1	Probe layer 2
AR platform Proximie®: overview	Describe Proximie®		
	What functions have you experienced on Proximie®?	Webinars	
		Streamed operation	Moderator
			Surgeon
			Assistant
			Participant
		Library	
		Remote training	
		Recording for portfolio	
	How much have you used Proximie®?	Frequency	
		Length of time	
	What was your electronic experience with Proximie®?	Ease of connection	
		Quality of resolution/sound	
		Tech required	Cost
			Complexity
AR platform Proximie®: relative advantages	What advantages does Proximie® have as a training tool?	Generally	
		During COVID-19	
		For specific types of trainees	Working pattern
			Personality type
			Learning style
	As a trainer...	…in comparison to other remote technologies?	Tech requirements
			Ease of use
			Team required
			'Webside manner'
		…in comparison to other in-person techniques?	Physical comfort
			Psychological comfort
			Experience
	As a trainee...	…in comparison to other remote technologies?	Tech requirements
			Ease of use
		…in comparison to other in-person techniques?	Experience
AR platform Proximie®:relative limitations	What limitations does Proximie® have as a training tool?	Generally	
		During COVID-19	
		For specific types of trainees	Working pattern
			Personality type
			Learning style
	As a trainer...	…in comparison to other remote technologies?	Tech requirements
			Ease of use
			Team required
			'Webside manner'
		…in comparison to other in-person techniques?	Physical comfort
			Psychological comfort
			Experience
	As a trainee...	… in comparison to other remote technologies?	Tech requirements
			Ease of use
		…in comparison to other in-person techniques?	Experience
Potential and further comments	How could Proximie® be improved?	From the trainee perspective?	
		From the trainer perspective?	
	Any further comments about the use of Proximie® or AR in general?		

## Results

Ten structured interviews were conducted, with a mean duration of nine (range: 5-30) minutes. Three participants were female and there was one senior house officer, four registrars, and three junior and two senior consultants (>10 years of experience). Six were born after 1980, and hence were considered 'digital natives' [3]. One was working from home at the time of the study. Refined code summaries are detailed below, with further reflections and quotations in Table 2.

1. Surgical AR as a substitute for hands-on learning

There was a consistent narrative that "surgery's surgery - so you have to do it". However, it was felt that engaging in streamed operations, "should count as the 'see one'", optimising rare theatre opportunities, and "allowing back and forth discussion with the operating surgeon", interaction considered crucial by participants in consolidating learning, and in nurturing longitudinal training relationships, from the safety of trainees' own homes. Far from solely mitigating COVID-19-related access issues, participants felt that, in certain circumstances, streamed procedures may even outperform physical observations (Table 2).

Using the underutilised option to put a camera "on your head when you're operating", in simulation or in vivo, could help move from "the see one" into "the practical part of surgery". This could enable retrospective video reflection or coaching or telementoring relationships, maintaining the human connection cherished in the apprenticeship model. The potential for recorded procedures to demonstrate competence through "video logbooks", feed into currently unsatisfactory work-based assessments, and, ultimately, support consultant credentialling, is exciting and requires further work.

2. Surgical AR and theoretical learning

The participants felt that learning "the steps, the procedural elements, and the theory" of surgery could be taken outside the operating theatre and that a curated, personal, comprehensive online multimedia library would be invaluable. Issues were raised around the engagement of learners and ensuring the quality of delivered remote teaching (Table 2).

3. Considerations specific to streamed procedures using Proximie®

**Figure 1:**
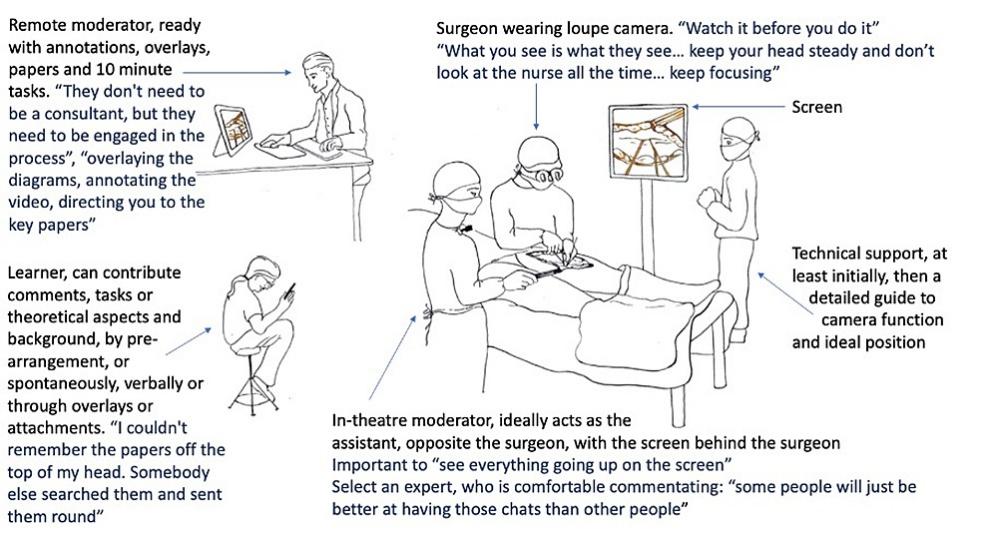
The recommended set-up for streaming procedures (as opposed to interactive telementoring, where the surgeon's view of the camera would necessarily differ)

In order to optimise the training potential of streamed, interactive operations for demonstration, a suggested team and layout were recommended (Figure 1) to form an appropriately briefed and prepared team containing a surgeon with a loupe-mounted camera, an "engaged" in-theatre moderator directing learners and answering questions, a remote moderator armed with 10-minute tasks “to fill in any gaps” including imaging, history and examination findings, mark-up exercises, anatomical overlays, relevant papers, task steps, and management protocols. Insights regarding technical orientation to the platform are presented in Table 2.

There were concerns around potential psychological "pressure on the operator" in operative streaming or avoiding it at the cost of missing out on a training opportunity, though there was a suggested benefit in developing resilience. The Proximie® platform is password-protected, end-to-end encrypted, and a robust consent process was followed; however, concerns regarding consent, confidentiality, data storage, and access were identified across multiple interviewees. The participants' reflections on protecting the psychological safety of the operator and patient are detailed in Table 2, as are the views on the need to institute a contingency plan should the operation fail to go as planned.

**Table 2:** Further comments and reflections relating to refined codes 1-4* *1. Surgical AR as a substitute for hands-on learning; 2. Surgical AR and theoretical learning; 3. Considerations specific to streamed procedures using Proximie®; 4. Medicolegal considerations in the use of technology in general (including Proximie®). The responses are summarised, with illustrative verbatim quotations AR: augmented reality

Refined codes 1-4: comments and reflections
Surgical AR as a substitute for hands-on learning
Interactive procedures may surpass in-person observation: in surgery with limited views, where your attention may be diverted ("as soon as you’re closing the donor site you’re not learning anything"), for rare procedures, and for trainees off-site "who may miss out on certain procedures just because they're not there". The ability to "interact" directly with the surgical team was deemed crucial in engaging learners: "we can all go on to YouTube and watch somebody raising a flap. But having a consultant talking through the process at the same time in real time… describing what's being done, explaining alternative techniques, explaining key papers, explaining the history behind it is an absolute gold mine". The AR functions were "useful to demonstrate particular nuances of anatomy when it comes to pedicle dissection, say, or flap planning". The ability for any observer to upload papers enabled the closure of the academic loop not usually possible mid-list. "That seems like the ideal marriage of giving you guys the technical insights into the operations and all the little things that have formed my opinion of why I do what I do". There is potential to relate to different learning styles: “reading the papers will probably stick with some people… and putting the line anatomical diagrams over the top… that will stick with some people". However, all felt that you cannot "take the practical part of surgery out of training". The potential for remote instruction of individuals in simulation or on patients was volunteered "as a training tool, rather than as a training tool for everyone" which could feed into a "video logbook"
Surgical AR and theoretical learning
It would be useful to store the streamed operative material for "visual revision" at a pertinent time, ideally as part of a fuller library resource with "online resources for literally everything you could possibly ever encounter in every exam. It shouldn't be another battle that you have to go and search out. It's just we're indoctrinated with the idea that you have to spend a vast amount of money on it yourself and it's not that easy to get hold of". Weekly webinars run via the platform were well-received, but not considered unique to the AR platform. The efficiency and equity of collaboration between departments were appreciated, as was the quality of the teaching received. "We can just open up our laptops and listen to what JP Hong's got to say about how he does perforator flaps… Twenty years ago, it would be a flight to South Korea to get that same experience". Engagement can be lacking in webinars compared to small group teaching. "For me, it's not interactive enough. You need to have that engagement in terms of the trainees being asked…questions, contributing, thinking about what's going on”. The lack of immediate interaction and feedback was perceived as challenging for trainers. "You don't get whether the feeling is positive or negative, or if you said something and everyone is staring at you". Formalising formative feedback was felt to be useful in maintaining and improving the standard and content of teaching. "All this feedback rubbish is actually quite useful, but we need to be a bit more clear in our feedback, not just 'that lecture was just tediously dull and shit and I haven't picked anything up from it'"
Considerations specific to streamed procedures using Proximie®
a) Platform orientation: though the layout "seemed pretty easy to use", it was felt that a formal orientation tutorial may be helpful, particularly for those less confident digitally. "If you were maybe 10 years older than me and less into computers I don't know how user-friendly it would be, but for me it was, it was perfectly user-friendly and I think that for anyone younger than me it would be". b) Considering the surgeon-perceived pressure on the operator was not felt by the participants is likely to impact the patient’s outcome, or to compromise the remote learners’ experience; however, there were concerns that having "the cameras on them and knowing that all their peers are watching them" could affect a registrar’s wish or opportunity to operate: "there'll be some trainees who hate that", which may forfeit them valuable training opportunities. Even consultants felt the pressure of operating with an invisible audience: "you're slightly less relaxed when everyone's watching… Just for a moment, having everyone watching transported me back to being an ST3 and doing my first ever flap anastomosis". c) Contingency planning: the in-theatre moderator was crucial, even more so when the operation was difficult, to safeguard the surgeon’s "bandwidth", and operative efficiency. "Relying on the operator fielding the questions and keeping the audience entertained whilst doing the operation is a big ask", with a risk that the surgeon may lose time and focus by having to look away from the operative field at the monitor to see what the listeners are demonstrating. When the operation was not going as planned, engaging with the streaming process became more difficult for the trainers. Here, the external moderator was felt to be essential to "fill in the gaps of silence". "In the very last one we did, which was a much harder operation, it was less easy to articulate all the bits of it…There wasn't enough bandwidth to go around". Introducing a debrief was suggested in these circumstances: "let's just all get on this call again now that we finished the operation… let's talk about all the hard bits and let's have that kind of debrief"
Medicolegal considerations in the use of technology in general (including Proximie®)
Patients "should have the same level of consent as publication in a journal of an identifying photo", which should exactly specify what the images are going to be used for. The rules of participation also need to be defined and monitored: “lots of people are logging in and they could even record it…it's patient information so that needs to be really tight" and access to and ownership of the footage needs to be pre-determined. "What happens with those videos now and what, you know, will there be a fee to watch them at any point in the future? And who owns them? And what can be done with them? We need to just drill down on exactly what's what with those"

## Discussion

The clinical and educational challenges thrown up by COVID-19 have been a rich incubator for technological solutions [5]. Despite the long-term interest in web-based educational strategy [6], the current crisis has initiated unprecedented streamlining of educational resources, between departments and via national associations [1], harnessing the collaborative mindset of emerging leaders [7]. In our department, engaging with AR has been well received by trainers and learners, facilitating regular teaching despite distancing measures through webinars, and operative involvement despite consultant-led operating and limited footfall, via remote operative streaming. It allows back and forth discussion with the operating surgeon, interaction considered crucial by participants in consolidating learning, and in nurturing longitudinal training relationships, from the safety of trainees' own homes. Far from solely mitigating COVID-19-related access issues, participants felt that, in certain circumstances, streamed procedures may even outperform in-person observation in the normal way: for small fields of view such as hand surgery, rare procedures, retrospective "visual revision" or situations where the trainee may be waylaid: “as soon as you're closing the donor site, you're not learning anything". Technology in a broader sense has streamlined referrals, clinics, multidisciplinary meetings, and administration [5,7], efficiencies potentially carving out more time for training, going forward.

In order to optimise the training potential of streamed, interactive operations for demonstration, a suggested team and layout were recommended (Figure 1). An appropriately briefed and prepared team were set up, consisting of a surgeon primed to "keep your head steady and focus"; an "engaged" in-theatre moderator directing learner attention and conversation, and answering questions; a remote moderator armed with a formulated educational patient- and procedure-specific materials and 10-minute tasks to flip to, “to fill in any gaps” such as imaging, history and examination findings, mark-up exercises, anatomical overlays, relevant papers, task steps, and management protocols. "That seems like the ideal marriage of giving you guys the technical insights into the operations and all the little things that have formed my opinion of why I do what I do". Technical support was also deemed essential, at least initially, with detailed instructions for use in their absence. The in-theatre moderator was crucial, even more so when the operation was difficult, to safeguard the surgeon's "bandwidth", and operative efficiency. "Relying on the operator fielding the questions and keeping the audience entertained whilst doing the operation is a big ask", with a risk that the surgeon may lose time and focus by having to look away from the operative field on the monitor to see what the listeners are demonstrating. A head-up display could improve interactive communication in the absence of an in-theatre moderator but would carry the known limitations of eye strain from a prolonged focus on a small screen, and short battery life [8].

The unquantified time and effort required both for moderators "to engage" sufficiently in preparing these sessions to optimise their value, and for trainees to focus on (and/or remotely contribute to) an entire case without distraction, requires acknowledgement. This may require protected time in working hours for trainees, and recognition of trainers' time and commitment, with formalised immediate and longer term feedback on, and responsibility for, their performance. Assessment of and training in 'webside manner', camera awareness and placement (the virtual "assistant" should be "on your shoulder" not pointing at the floor) and strategies to mitigate any physical strain for the surgeon due to keeping the view steady may be helpful, with pre-session training, post-session debriefs, drop-in support, and iterative sharing with others described in the literature to enhance this [9]. To achieve this efficiently, and to continue to benefit from a collaborative approach, entails wider discussions around structured and collegiate approaches to standardising and optimising training, recognising and giving responsibility to trainers, co-constructing learning opportunities and curricula in a learner-centred model, and overcoming siloed learning by sharing strategies and experiences grounded in lived experience. 

Using the already existing, but underutilised, option to put a camera on your head when you are operating, in simulation or in vivo, could move from "the see one" into "the practical part of surgery". This may enable retrospective video reflection or coaching [10] or provide a platform for remote real-time telementoring relationships [11] while maintaining the human connection so cherished in the apprenticeship model. The potential for recorded procedures to demonstrate competence through "video logbooks", feed into currently unsatisfactory [12] trainee work-based assessments and, ultimately, to support credentialling and continuing professional development for consultants is exciting and requires further work exploring the complexities of reliable and valid assessment methodology [13].

Augmenting the interactive streaming by developing the library function, with customisable, quality-assured resources, succinct, curated, and adhering to fellowship standard, was seen as having the potential to cut through training inequities: inequities derived from working patterns or regional differences, service expectations in the theatre, financial disparities, tech-savviness, time constraints, or the ability to travel: "20 years ago, that would be a flight to South Korea to get that same experience", at least in relation to the observational "see one", "visual revision", and the steps, procedural elements, and theory of surgical training.

Participants felt that protecting psychological safety [14] is paramount, both of the trainers (moderator and surgeon) and the patient. We discussed the need for offsetting the unease of the moderator in fielding questions in a large group of strangers by limiting the number and nature of attendees and using the external moderator as gatekeeper. There were concerns around the potential psychological "pressure on the operator" in operative streaming or the stress of avoiding it at the cost of missing out on a training opportunity: "there'll be some trainees who'll hate that", though there was suggested benefit in developing resilience. This perceived pressure on the operator was not felt by the participants, which is likely to impact the patient's outcome, or to compromise the remote learners' experience; however, concerns regarding consent, confidentiality, data storage, and access were clearly identified across multiple interviewees. The Proximie® platform is password-protected, end-to-end encrypted, and a robust consent process was followed. This was informed, specific to streamed procedures, differentiated and watertight for the sakes of the patient and of the clinicians, in a medium where it can be easy to lose control of data [8]. Understanding that patients may wish to access their data, across a range of operative outcomes, is important to pre-empt, and it could be made available to them on application, with ownership, distribution, and access comparable to traditional medical photography.

Like any burgeoning innovation, pre-empting issues in educational technology is crucial, to sidestep avoidable criticism or slowing of forward momentum. Many of the points discussed are not limited to AR platforms but applicable to multiple educational technological and collaborative initiatives. The amount of initial training for students to be comfortable in using web-based tools is often underestimated [15] and may require learner needs' assessment to appropriately orientate those less technologically adept than others. Established engagement and knowledge consolidation strategies could be considered, such as remote or in-person "interactive" small group debriefs [15], pre- and post-educational intervention MCQs, topic selection, real-time co-annotation, and polls [2]. More agile options of validating and rating resources and submissions to open fora than traditional journal-style peer-review could be introduced to help with peer curation. Finally, appropriately reliable and encrypted network access, and scaling to variable bandwidths and accessible browsers should be prioritised, to maintain audiovisual quality.

This study is limited to the experience of 10 interviewees, which may not represent international, regional, or subspecialty variations or the wider surgical team and patient. However, discussing the general themes, common narratives, and different perspectives may inform others using AR and technology in general in surgical education, as we all progress in collaboration. Furthermore, this may help establish important aspects of standardisation in such a platform and enable calibrations for broader applications in the future.

## Conclusions

Harnessing novel technologies in surgical education represents an exciting opportunity, fast-tracked by the COVID-19 pandemic, but applicable beyond it. Though this study had a small sample size, its results suggest that AR platforms may offer uniquely interactive remote educational experiences in surgical training. The streamed procedures and webinars were well received in our department, and there is potential to develop these further, along with more hands-on training tools, such as coaching and telementoring, going forward.
